# Clinical trials of stem cell-based therapies for pediatric diseases: a comprehensive analysis of trials registered on *ClinicalTrials.gov* and the ICTRP portal site

**DOI:** 10.1186/s13287-022-02973-2

**Published:** 2022-07-15

**Authors:** Yinwen Ji, Chuan Hu, Zuxing Chen, Ying Li, Jiayong Dai, Jin Zhang, Qiang Shu

**Affiliations:** 1 National Clinical Research Center for Child Health, The Children’s Hospital, Zhejiang University School of Medicine, Hangzhou, 310052 People’s Republic of China; 2 Key Laboratory of Musculoskeletal System Degeneration and Regeneration Translational Research of Zhejiang Province, Sir Run Run Shaw Hospital, Medical College of Zhejiang University, Hangzhou, 310016 People’s Republic of China; 3 Department of Orthopedics Surgery Shengli Clinical Medical, College of Fujian Medical University, 134 Dongjie Road, Fuzhou, 350001 People’s Republic of China; 4 College of Chemical Engineering, Fuzhou University, 2 Xueyuan Road, Fuzhou, 350108 People’s Republic of China

**Keywords:** Stem cell, Children, Pediatric diseases, *ClinicalTrials.gov*, ICTRP, Trial registration

## Abstract

**Background:**

Research on clinical trials that employ stem cells to treat children’s diseases is limited. The clinical trial registry database provides a unique window to us to get known about clinical trial researches with different statuses. However, few studies aimed to perform a comprehensive and thorough analysis of those registered trials in the aforementioned field based on *ClinicalTrials.gov* and the ICTRP portal site.

**Methods:**

Our study covered the clinical researches about stem cell therapy enrolling subjects aged under 18 years old registered on ClinicalTrials.gov and WHO ICTRP before May 18, 2021. A cross-sectional study was implemented to comprehensively describe and analyze the included trials that met the criteria. Results were available on ClinicalTrials.gov, and publications related to the included trials were identified. All analyses were performed utilizing the SPSS 25.0 software.

**Results:**

Eventually, 202 clinical trials were included and evaluated. The participant number of trials tended to be small; 71.3% were enrolled < 50. And 93.5% of the subjects were without gender restrictions. Till May 2020, 112 trials had been preliminary completed, of which only 39 trials had published papers or uploaded results. Most (73.6%) of 186 interventional trials were in phase 1 and phase 2, where 131 (70.4%) trials were conducted without masking, and 26.3% trials were randomized; 55.4% trials were performed single group assignment. Of 16 observational trials, case-only/series took up 37.5%. Hematopoietic stem cells (37.1%) and mesenchymal stem cells (36.1%) were mostly employed, while umbilical cord blood (UCB)-derived cells (24.3%) and bone marrow (BM)-derived cells (20.8%) were the major sources.

**Conclusions:**

This study provided an overall picture of utilizing stem cells for treatment and management of childhood diseases. Since clinical trials in this area are insufficient in quantity and quality, there is an urgent need of larger, better-designed trials. Increased investment in clinical research of stem cell treatment products should be carried out to achieve the transformation of results as soon as possible. Moreover, it is important to optimize the management of the registration platform and shorten the time it takes for research results to be published.

## Introduction

Every child has a unique opportunity to reach his or her full potential by growing up to be a healthy adult, and children’s health is closely related to all aspects of the growth and development of children. Although significant improvements have been made in children’s survival, nutrition, and education over recent decades, progress on indicators of children’s health and well-being across the Sustainable Development Goal (SDGs) is currently at a standstill [1]. The steady increase in the incidence of many diseases among children has led to a gradual increase in the burden of childhood disease and health care [2–5]. As is well known, conducting clinical trials in children can help researchers to discover the best way to treat pediatric diseases, thus further dramatically improving their health care. Given the scientific, ethical, and practical difficulties, conducting clinical trials in children is commonly considered challenging. According to *Clinical Trials.gov* (https://clinicaltrials.gov/. Assessed August 26, 2021), only 19.9% (77,334/387966) of the registered trials included only children as study subjects. About half of all pediatric trials remain unfinished or unpublished after trial completion [6], higher quality and larger quantity clinical trials in children are needed to expand our understanding of the treatment of pediatric diseases.

At an age of diagnosis and treatment technology develop and innovate rapidly, immune therapy, gene therapy, and stem cell therapy represent the cutting-edge research area. Stem cell therapy refers to the transplantation of healthy stem cells into a patient or into the body itself to repair diseased cells or rebuild functioning cells and tissues. According to the Stem Cell Market Size Analysis (2019–2025), the number of diseases that stem cells can treat increased by 300% between 2005 and 2013 [7]. As an emerging technology, stem cell therapy has been extensively used in the prevention and treatment of cardiovascular disease, cancer, spinal cord injury, Parkinson’s disease, immune disorder, and other diseases [8–14]. In the future, with the development of stem cell technology, the number of treatable diseases will continue to increase, whereas there is little known about clinical research on stem cells in treating pediatric diseases.

As a comprehensive and transparent reporting platform, clinical trial registration system together with clinical research methodology constitutes an external guarantee system to ensure the authenticity of clinical trials, so that the implementation of clinical trials has rules to follow and the influence of all artificial or non-human bias on the authenticity of clinical trials can be reduced as far as possible [15]. *ClinicalTrials.gov* was jointly developed by the National Library of Medicine, the affiliated unit of the National Institutes of Health (NIH), and the Food and Drug Administration (FDA) of the United States in 1997 and started operation in 2000 [16]. As one of the most commonly used clinical trial registration platforms [17], a total of 382,313 clinical studies from 50 states and 220 countries were registered on the website up to July 6, 2021, including a number of trials on stem cell therapy in children. Additionally, the World Health Organization (WHO) International Clinical Trials Registry Platform (ICTRP, https://trialsearch.who.int/Default.aspx) synthesizes an additional 17 registered sources worldwide to provide more comprehensive clinical trial data. Therefore, we retrieved and analyzed all of these trials focused on stem cell therapy for pediatric diseases registered on *ClinicalTrials.gov* and WHO ICTRP to assess the characteristics and the trends in this field.

## Materials and methods

### Reporting Guideline

This belonged to a cross-sectional study, and it followed the Strengthening the Reporting of Observational Studies in Epidemiology (STROBE) reporting guideline [18].

### Retrieval and screen of relevant registered trials

We carried out a cross-sectional study of registered trials about using stem cell technology in the treatment of childhood diseases based on the *ClinicalTrials.gov* database and the ICTRP portal site, and we selected the clinical trials registered before May 18, 2021. The trials were obtained from the website through choosing the advanced search function, with the search term “stem cell” for “intervention/treatment”, checking “(birth-17)” for “Age Group” on *ClinicalTrials.gov* and we searched on the ICTRP with "stem cell" as the keyword. Subsequently, some vital data were exported into Excel, and then, we manually scanned the title of each trial for further discrimination. Furthermore, we retrieved complete research details for every potentially qualified trial and independently evaluated the inclusion. As this study was predetermined as a purely pediatric trial from the very beginning, we only included studies where all subjects were less than 18 years old, and trials not investigating stem cell approach in children's diseases were excluded. The research did not involve human subjects; thus, institutional review board (IRB) and written consent were not required.

### Data extraction

The following information was extracted from the Tabular View of *ClinicalTrials.gov* and the ICTRP, including (1) Tracking Information: first submitted date, first posted date, study start date/date enrollment, primary completion date; (2) Descriptive Information: brief title/public and scientific title, detailed description, study type, study phase, study design including interventional study (allocation, intervention model, masking, primary purpose) and observational study (model, time perspective), condition; (3) Recruitment Information: estimated enrollment/target size, sex, ages/inclusion age_min_ and age_max_, location countries; (4) Administrative Information: NCT number/Trial ID and Secondary ID, data monitoring committee (DMC), study sponsor/primary sponsor, collaborators. Other cell information was extracted from research details, *e.g.,* origins (autologous or allogeneic), source organs, cell types, routes of administration, dispose of stem cells and whether to combine other treatments by our manually reviewing of each trial’s record.

### Search for corresponding publication of included trials and determine the time to publication

The “publications of results” and “publications automatically indexed to this research by *ClinicalTrials.gov* identifier (NCT Number)” field in the *ClinicalTrials.gov* database was retrieved and used to search for potentially matched publications. Meanwhile, we manually utilized NCT numbers/Trial ID or brief/public and scientific titles to search for corresponding publications on PubMed and Google Scholar by July 27, 2021. Reviews, meta-analysis, study protocols, and other irrelevant publication without research results were excluded. If more than one publication was obtained for the same registry trial, we selected the earliest article reporting primary outcome results. We determined the publication time by calculating period (in months) between principal completion date of the included studies and publications.

### Statistical analysis

The sponsors were classified as non-industry (non-profit organization (NPO) or university and hospital), industry or other sponsors. Categorical data were reported as frequency and percentage. Continuous variables were reported as median (interquartile range, IQR). The differences in categorical variables between groups were compared using the chi-square test or Fisher exact test. In order to analyze cumulative publication rates after primary trial completion, the Kaplan–Meier analysis method was applied, and those primary trials completed after May 18, 2020, were excluded because of the completed trials needed sufficient time to be published. All analyses were performed using the SPSS 25.0 software (IBM, USA). A *P* value < 0.05 (two-sided) was considered significant.

## Results

### Screening and Included trials

**Fig. 1:**
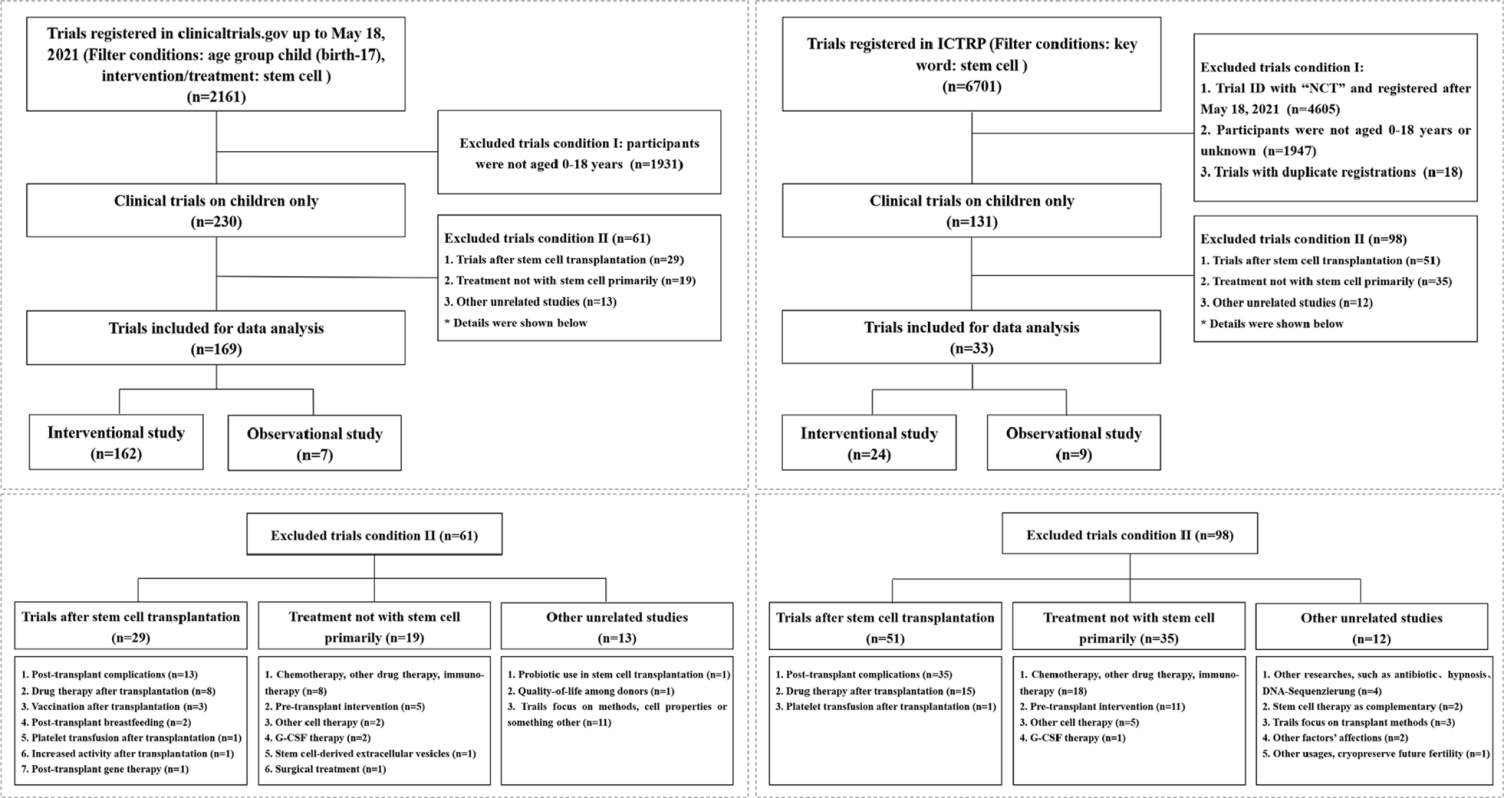
Flowchart of selection on *ClinicalTrials.gov* and ICTRP up to 18th May 2021. Abbreviation: G-CSF, granulocyte colony stimulating factor

We identified 8862 registered trials on *ClinicalTrials.gov* and ICTRP in the original retrieval. After excluding trials with participants more than 18 years old and trials which were not related to stem cell therapy for pediatric diseases, 202 trials were eventually included for analyses (Fig. 1).

### Basic characteristics of included clinical trials

As shown in Table 1, during the 4 time-subsets (Prior to 2003, 2003–2007, 2008–2012, 2013–2017, 2018-mid2021), the number of registered clinical trials were presented a gradual increasing trend (6, 18, 45, 60, 73), of which, the vast majority of trials (*n* = 178, 88.1%) were registered after 2007. In initial completed clinical trials, most of the studies spanned no more than 36 months (*n* = 71, 35.2%), while only a few studies lasted more than 72 months (*n* = 18, 8.9%). The bulk of these trials enrolled < 50 (*n* = 144, 71.3%); only 11 trials (5.5%) recruited 200 or more participants. Of the qualified trials, 133 (65.8%) were single centers, 39 (19.3%) were conducted in single country-multicenter, and only 16 (7.9%) were scattered multiple centers across multiple countries. 69 trials (34.2%) were in the completed state, followed by the recruiting state (*n* = 45, 22.3%), 11 trials (5.5%) were suspended, 8 trials (4.0%) were terminated, and 8 trials (4.0%) were withdrawn. Almost all of the subjects (*n* = 189, 93.6%) were not limited by gender of the participants, merely thirteen of the studies only included males. Over half of the studies 117 (57.9%) provided DMC. A total of 112 (55.5%) have been completed up to May 2020, among them, only 39 have shown publications (including results).

**Table 1:** General characteristics of 202 included trials

Characteristics	Number/value	Percentage (%)
*Submit year*		
Prior to 2003	6	3.0
2003–2007	18	8.9
2008–2012	45	22.3
2013–2017	60	29.7
2018-mid-2021	73	36.1
*Study period*		
L ≤ 36 m	71	35.2
36 < L ≤ 72 m	32	15.8
72 m < L	18	8.9
NA	81	40.1
*Actual/estimated enrollment*		
*n* < 50	144	71.3
50 ≤ *n* < 100	30	14.9
100 ≤ *n* < 200	13	6.4
*n* ≥ 200	11	5.5
NA	4	2.0
*Location*		
Single center	133	65.8
Single country-multicenter	39	19.3
Multicountry-multicenter	16	7.9
NA	14	6.9
*Recruitment status*		
Completed	69	34.2
Recruiting	45	22.3
Active, not recruiting	18	8.9
Not yet recruiting	10	5.0
Terminated	8	4.0
Withdrawn	8	4.0
Suspended	11	5.5
*Unknown*	33	16.3
Participant gender		
Male and female	189	93.6
Male only	13	6.4
DMC		
No	45	22.3
Yes	117	57.9
NA	40	19.8
Publication (including results)*		
Primary completion and publication (including results)	39	19.3
Primary completion but not publication (including results)	73	36.1
Uncompletion	90	44.6

* May 2020 as the deadline. NA, not available

### Detailed characteristics of the included clinical trials

**Fig. 2:**
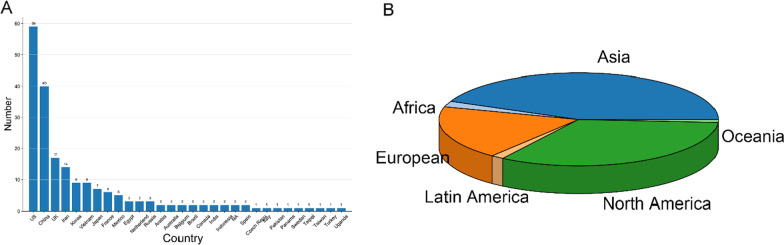
Distribution and analysis of countries and continents. **A** Bar-graph analysis of distribution by countries. **B** Pie-chart analysis of distribution by continents

Analyzed results by sponsoring countries are shown in Fig. 2A. The USA topped the list with 59 clinical trials, 20 more than second-place China, and the UK occupied the third place. Figure 2B exhibited a pie-chart by continental area, and distribution of the continents was consistent with countries, with Asia at the top (43.6%), followed by North America (33.2%) and Europe (18.8%).

**Fig. 3:**
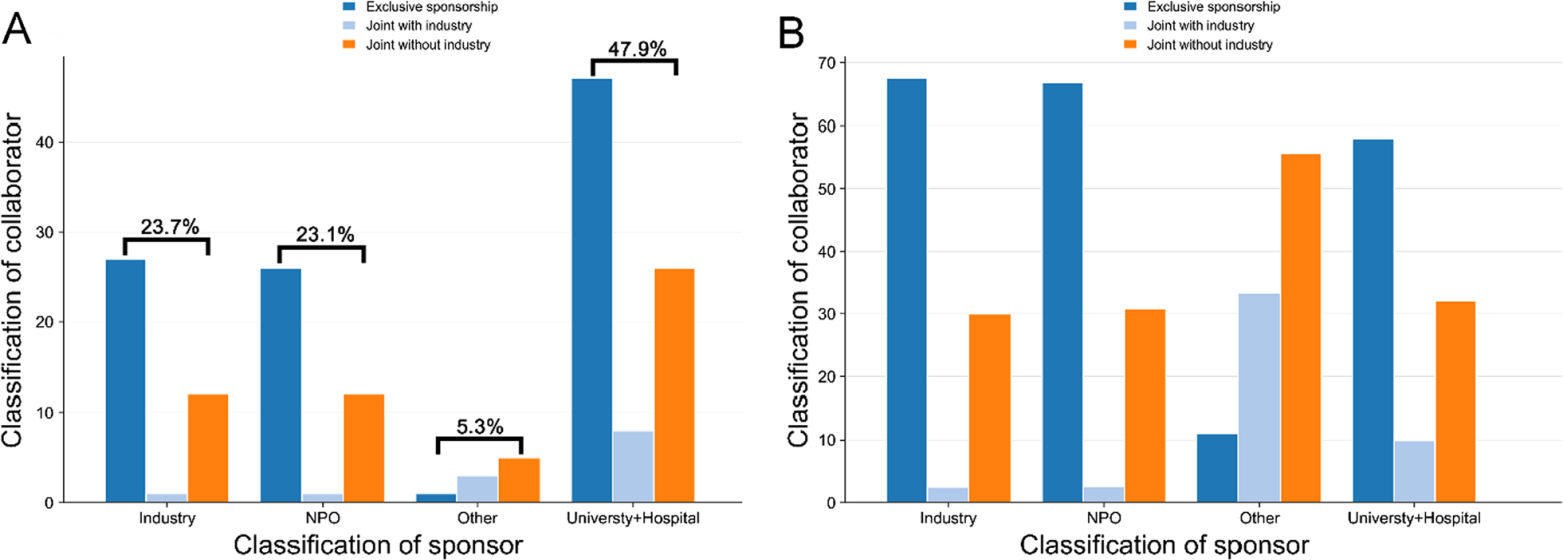
The distribution of collaborators in trials initiated by different sponsors. Data from 169 trials registered on *ClinicalTrials.gov*. **A** Number of collaborators trials initiated by different sponsors. **B** Percentage of collaborators trials initiated by different sponsors

Figure 3 shows most (47.9%) of trials were sponsored by university and hospital, and the number of trials funded by industry and NPO was almost the same. Moreover, except for the “Other” group, three groups were listed as follows: exclusively-sponsored accounted for the most, sponsored by other organization joint without industry accounted for the next, and industry cooperation accounted for the least.

**Fig. 4:**
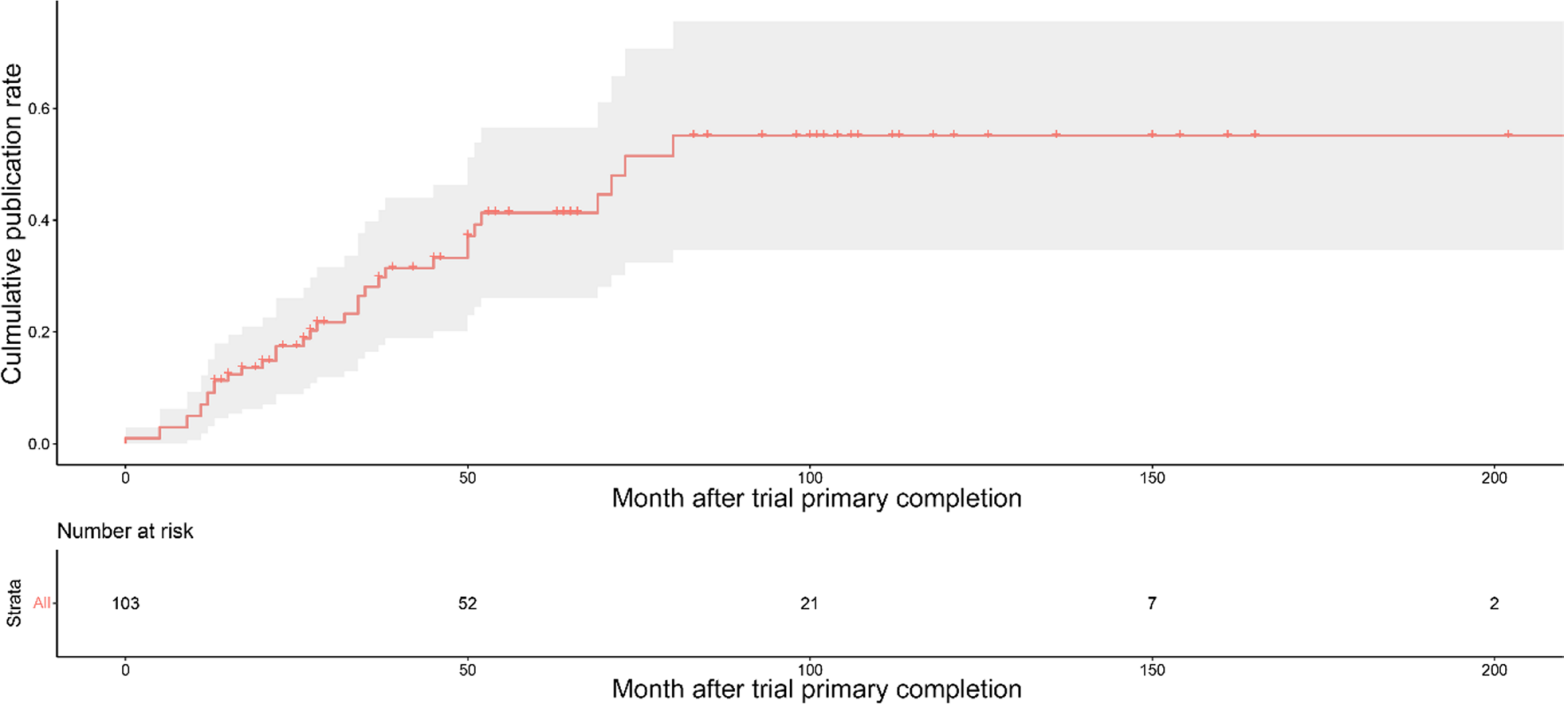
Cumulative publication rate curve since the initial completion of the clinical trials

Overall, the 5-year cumulative percentage of publication after trial completion was 33.9%. After the clinical trials were completed, the cumulative publication rate indicated a gradually increasing trend, with publication rates of 8.7% at 12 months, 16.0% at 24 months, 24.5% at 36 months, and 28.3% at 48 months, respectively (Fig. 4).

The characteristics of North America/Europe and other countries are demonstrated in Table 2. After analysis, we identified that compared with other countries, Europe and North America started trials earlier than other countries, and the number of trials in non-European and North American countries showed a trend of gradual growth as well as surpassed that of European and North American countries during the period of 2013–2017. Moreover, the proportion of multicenter trials was also significantly higher. In terms of information on the origin of stem cells, the European and North American studies disclosed more complete information, while 26.8% of the studies conducted by other countries did not disclose this information in detail. In addition, the proportion of Occidental trials that disclosed detailed product information was significantly higher than trials conducted by other countries, and the proportion of combined treatments was also significantly higher. In non-European and American countries, more than half of the trials were sponsored by universities and hospitals.

**Table 2:** Characteristics of North America/European and Other countries

		North America and European (*n* = 105)	Other (*n* = 97)	χ2/Fisher	P
Submitted year	Prior to 2003	6 (5.7%)	0 (0)	36.324	< 0.001
	2003–2007	18 (17.1%)	0 (0)		
	2008–2012	29 (27.6%)	16 (16.5%)		
	2013–2017	28 (26.7%)	32 (33.0%)		
	2018-mid2021	24 (22.9%)	49 (50.5%)		
Publication	No	83 (79.0%)	78 (80.4%)	0.058	0.810
	Yes	22 (21.0%)	19 (19.6%)		
Sponsor	University and Hospital	42 (40.0%)	62 (63.9%)	11.738	0.008
	Industry	26 (24.8%)	16 (16.5%)		
	NPO	28 (26.7%)	14 (14.4%)		
	Other	9 (8.6%)	5 (5.2%)		
Location	Multicountry-multicenter	16 (15.2%)	0 (0)	20.152	< 0.001
	Single center	62 (59.0%)	71 (73.2%)		
	Single country-multicenter	23 (21.9%)	16 (16.5%)		
	NA	4 (3.8%)	10 (10.3%)		
Estimated Enrollment	< 50	79 (75.2%)	65 (67.0%)	7.151	0.128
	50 ≤ *n* < 100	12 (11.4%)	18 (18.6%)		
	100 ≤ *n* < 200	6 (5.7%)	7 (7.2%)		
	≥ 200	4 (3.8%)	7 (7.2%)		
	NA	4 (3.8%)	0 (0)		
Gender	All	98 (93.3%)	91 (93.8%)	0.019	0.889
	Male only	7 (6.7%)	6 (6.2%)		
DMC	No	23 (21.9%)	22 (22.7%)	3.234	0.199
	Yes	66 (62.9%)	51 (52.6%)		
	Not provided	16 (15.2%)	24 (24.7%)		
Study type	Interventional	100 (95.2%)	86 (88.7%)	2.992	0.084
	Observational	5 (4.8%)	11 (11.3%)		
Named product	No	83 (79.0%)	89 (91.8%)	13.873	0.011
	Yes	22 (21.0%)	8 (8.2%)		
Combination therapy	No	67 (63.8%)	79 (81.4%)	7.825	0.005
	Yes	38 (36.2%)	18 (18.6%)		
Autologous/allogeneic	Allogeneic	46 (43.8%)	37 (38.1%)	8.144	0.043
	Autologous	46 (43.8%)	33 (34.0%)		
	Allogeneic + autologous	0 (0)	1 (1.0%)		
	NA	13 (12.4%)	26 (26.8%)		

* NA, not available

### Methodological quality of the included clinical trials

Of all the 202 included trials, 186 (92.1%) were interventional studies. Among these 186 interventional trials, phases of trials were presented as follows: phase 1 (26.3%), phase 1 phase 2 (24.2%), phase 2 (23.1%), phase 3 (5.4%), phase 2 phase 3 (3.2%), and phase 4 (1.1%). 26.3% were randomized, 21.5% were non-randomized, and the rest 52.5% were not available. There were 103 (55.4%) trials containing single group assignments, followed by 57 (30.7%) were parallel assignments. 70.4% were not masked, only 16.7% of the included trials used masking. The vast majority of primary purpose in the interventional trials was to treatment (81.2%) and prevention (3.8%). Of the sixteen observational studies, case-only/series and cohort accounted for 6 (37.5%) and 3 (18.8%), respectively. Meanwhile, 4 (25.0%) of these trials were prospective (Table 3).

**Table 3:** Study design of included trials

Study type	Study design	Number (*n*)	Percent (%)
Interventional			
*Phases*			
	Phase 0	5	2.7
	Phase 1	49	26.3
	Phase 1 Phase 2	45	24.2
	Phase 2	43	23.1
	Phase 3	10	5.4
	Phase 2 Phase 3	6	3.2
	Phases 4	2	1.1
	NA	26	14.0
*Allocation*			
	Randomized	49	26.3
	Non-randomized	40	21.5
	NA	97	52.2
*Intervention model*			
	Single group assignment	103	55.4
	Parallel assignment	57	30.7
	Crossover assignment	7	3.8
	Factorial assignment	1	0.5
	NA	18	9.7
*Masking*			
	None (Open Label)	131	70.4
	Single	9	4.8
	Triple	9	4.8
	Quadruple	8	4.3
	Double	5	2.7
	NA	24	12.9
*Primary purpose*			
	Treatment	151	81.2
	Prevention	7	3.8
	Basic science	2	1.1
	Device feasibility	1	0.5
	Supportive care	1	0.5
	Other	1	0.5
	NA	23	12.4
Observational			
*Observational model*			
	Case-Only/series	6	37.5
	Cohort	3	18.8
	Other	3	18.8
	NA	4	25.0
*Time perspective*			
	Prospective	4	25.0
	Other	2	12.5
	NA	10	62.5

*NA, not available

### Description of stem cell and diseases categories in included clinical trials

**Fig. 5:**
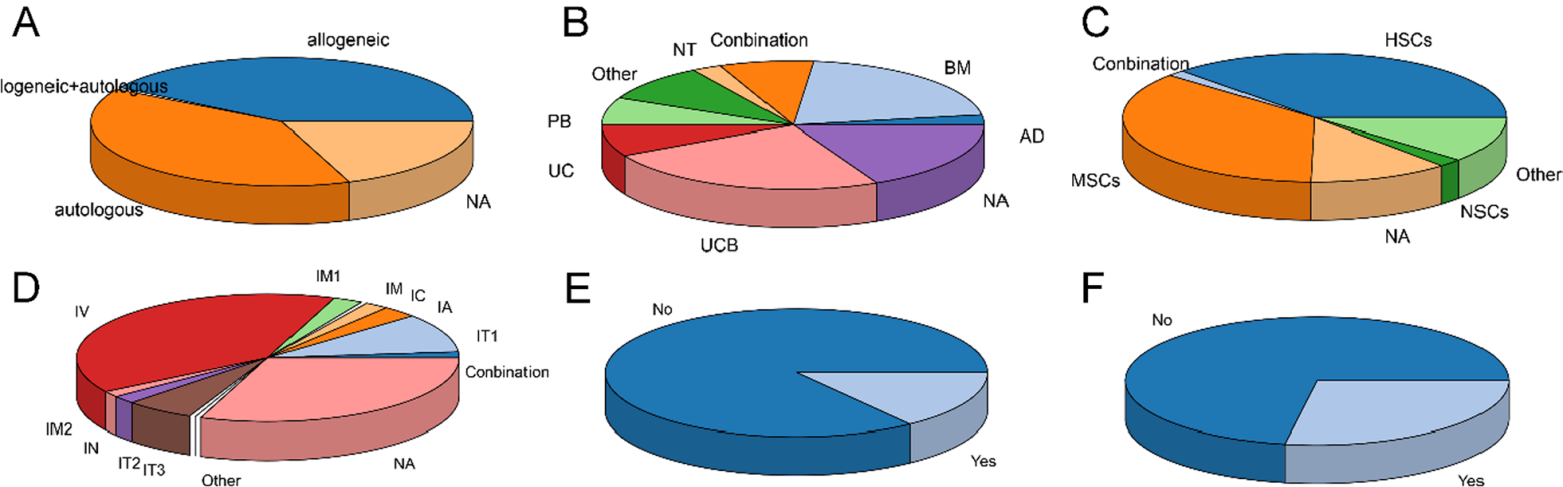
Distributions of origin, source organ, cell type, route of administration, specific product mentioned, and combination therapy of cell therapy. **A** Distribution of origins (autologous and allogeneic). **B** Distribution of source organs (BM, PB, UCB, UC, NT, AD, combination and other). **C** Distribution of cell types (NSCs, MSCs, HSCs, other and combination). **D** Distribution of administration routes (IC, IT1, IT2, IV, IA, IM1, IM2, IN, and combination). **E** Distribution of specific product names mentioned. **F** Distribution of combination therapy. BM: bone marrow, PB: peripheral blood, UCB: umbilical cord blood, UC: umbilical cord, NT: neural tissue, AD: adipose tissue, NSCs: neural stem cells, MSCs: mesenchymal stem cells, HSCs: hematopoietic stem cells, NA: not available, IC: intracranial, IT1: intrathecal, IT2: intratracheal, IV: intravenous, IA: intraarterial, IM1: intramyocardial, IM2: intramuscular, IN: intranasal

**Fig. 6:**
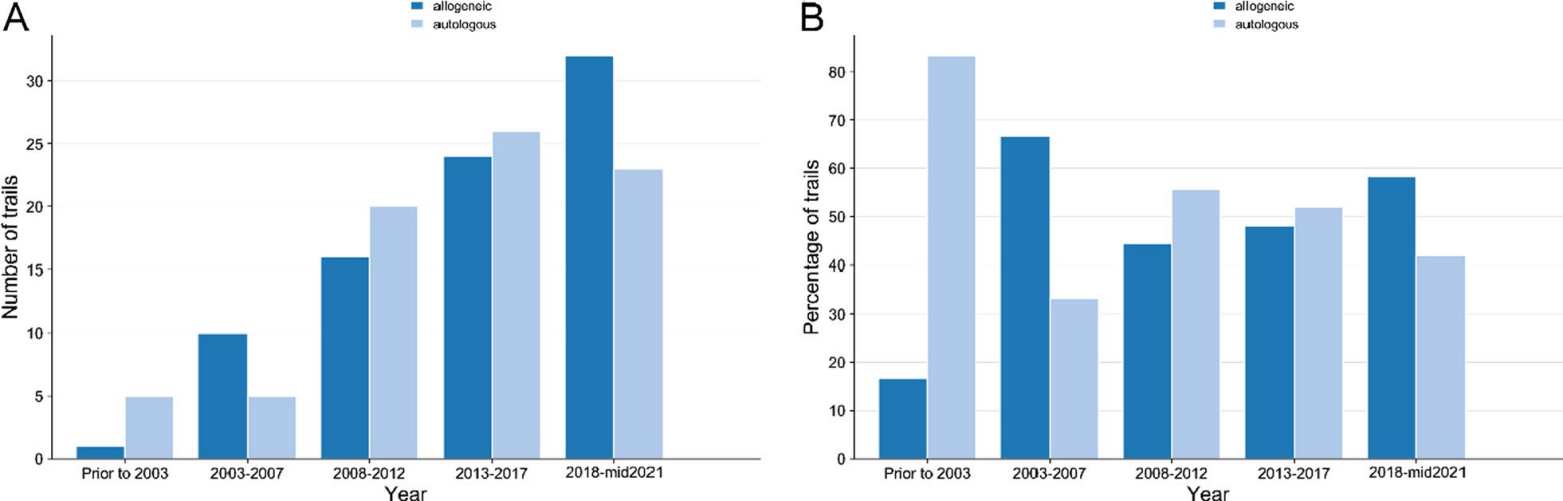
Number and percentages of autologous and allogeneic stem cells in different study years. **A** Number of autologous and allogeneic stem cells in different study years. **B** Percentages of autologous and allogeneic stem cells in different study years

We categorized the total eligible studies by the source of origin (autologous or allogeneic) of the stem cell used (Fig. 5A). It was revealed that the distribution trends of allogenic and autologous cells were mainly the same (41.1% and 39.1%, respectively). Further, as shown in Fig. 6, the distributions of autologous and allogeneic stem cells in various years indicated a slight fluctuation. In the stage 2003–2007, the proportion of allogeneic stem cells was 66.7%, but that number has leveled off since 2008. Moreover, in terms of source organs and cell types, there was an uneven distribution (Fig. 5B). We found that umbilical cord blood (UCB)-derived cells were used mostly, reaching 24.3%, followed by bone marrow (BM)-derived cells (20.8%), while adipose tissue and neural tissue were the lowest proportion, only 2.5% respectively. In addition, the sort of cell type suggested that hematopoietic stem cells (HSCs) made up the largest proportion, reaching 37.1%, followed by mesenchymal stem cells (MSCs) (36.1%), whereas some of the studies were conducted simultaneously with two or more cell types, which we attributed to the combination, accounting for 1.5% (Fig. 5C). Alternatively, in the route of administration, intravenous was 39.6%, ranking first, significantly higher than other methods of administration (Fig. 5D). As a result of clustering by whether combined with other treatments and whether mentioned products (Fig. 5E and F), we identified the majority of studies still did not involve cell products (85.1%) and combination therapy (72.3%).

**Fig. 7:**
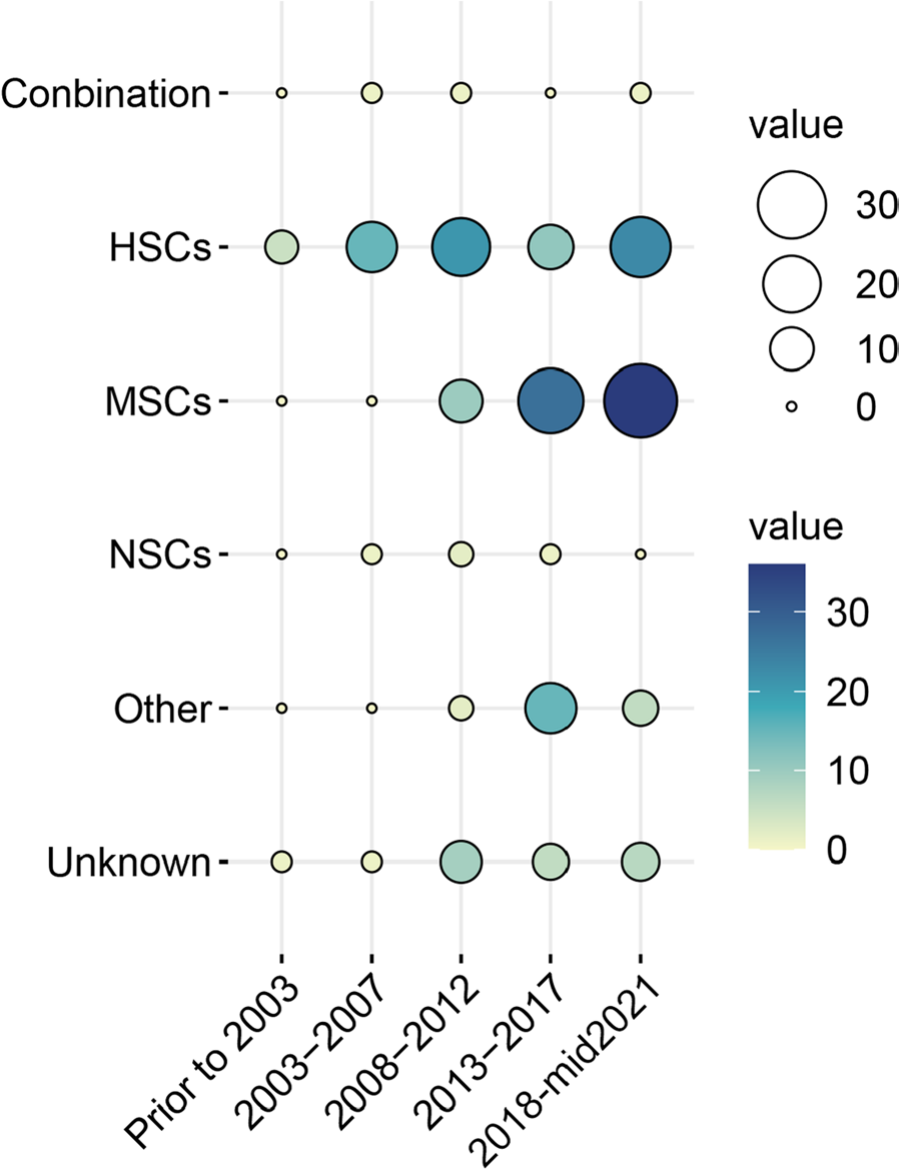
Transition analysis of clinical trials of cell therapy by cell type

The classification of cell types in different periods is shown in Fig. 7. It was not difficult to find the number of trials on MSCs presented a trend of gradual increase, while the number of trials on other cell types presented a relatively-stable trend. Specifically, the period 2008–2012 seemed to be a critical watershed in the MSCs, and from this period, trials focused on MSCs increased significantly and stabilized. In addition, HSCs belonged to a cell type that had been studied extensively from an early stage and remained stable over subsequent periods of time.

**Fig. 8:**
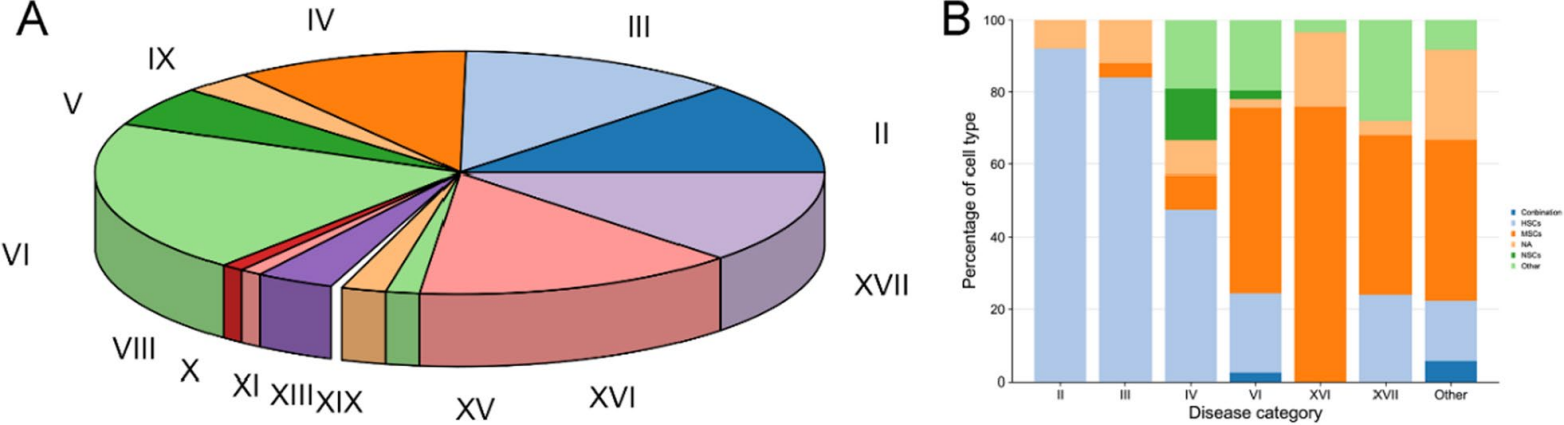
Distribution of disease and cell types in different diseases. **A** Distribution of the percentage of trials for different diseases category. **B** Distribution of the percentage of cell types used in trials for different diseases category. Involved diseases were classified by ICD-10: II Neoplasms, III Diseases of the blood and blood-forming organs and certain disorders involving the immune mechanism, IV Endocrine, nutritional and metabolic diseases, IX Diseases of the circulatory system, V Mental and behavioral disorders, VI Diseases of the nervous system, VIII Diseases of the ear and mastoid process, X Diseases of the respiratory system, XI Diseases of the digestive system, XIII Diseases of the musculoskeletal system and connective tissue, XIX Injury, poisoning and certain other consequences of external causes, XV Pregnancy, childbirth and the puerperium, XVI Certain conditions originating in the perinatal period and XVII Congenital malformations, deformations and chromosomal abnormalities

In 202 trials, 14 disease categories were included, and the top six disease categories were VI (Diseases of the nervous system), XVI (Certain conditions originating in the perinatal period), XVII (Congenital malformations, deformations and chromosomal abnormalities), III (Diseases of the blood and blood-forming organs and certain disorders involving the immune mechanism), II (Neoplasms) and IV (Endocrine, nutritional and metabolic diseases), in that order (Fig. 8). These six disease categories accounted for 82.3% of all trials (Fig. 8A). Then, we further analyzed the cell types used in the trials for each of the six disease categories. For trials focused on the II, 92.0% of the trials used HSCs, while the proportion of the trials for XVI was 0 (Fig. 8B). As another widely-studied cell type, MSCs was relatively high in trials for VI, XVI and XVII (51.2%, 75.9% and 44.0%, respectively), as displayed in Fig. 8.

## Discussion

This study conducted an all-around evaluation of the registered clinical trials about using stem cell in pediatric diseases on *ClinicalTrials.gov* and WHO ICTRP. In the retrieval process, we found that only around one out of ten trials just for children under the age of 18. Obviously, it is still very lacking in studies about utilizing stem cells in childhood illnesses. Therefore, we recommend that researchers expanded scope of the crowd in future clinical trials to obtain more clinical data of stem cell therapy in childhood diseases treatment area. In our analysis, roughly one-third of recruitment status were completed, and status of suspended, terminated, or withdrawn accounted for 13.5%. Among which, lack of patients, decision of sponsor, resignation of principal investigator, insufficient evidence of efficacy, protocol changes, and COVID-19 became main reasons. Most trials (*n* = 144, 71.3%) tended to be small sample size studies (< 50), and 57.9% were set up DMC. Most trials were sponsored by universities and hospitals, a great majority of trials were sponsored by Asia, North America and Europe. Notably, 133 of 202 trials were conducted in a single center, only 7.9% were multicountry-multicenter. A total of 39 of 112 clinical trials marked primary completion date by May 2020 have released results or published in peer-reviewed journals. Up to 18th May, 2021, most study period of clinical trials had primary completed was within 36 months. An overwhelming number of trials took interventional design, and only 16 were observational. More than half of the interventional trials were in phase 1 or 2, without providing whether they were randomized. And a large quantity of trials were only with single group assignments and non-masking. Most observational trials did not demonstrate time perspective. According to ICD-10, our survey covered 14 diseases, with a large number of treatments for childhood diseases using HSCs and MSCs.

Public registration of clinical trials, on the one hand, could protect participants from unnecessary duplication of research. On the other hand, this could enhance transparency and overcome the publication and selective results reporting bias [19]. As early as 2004, the International Committee of Medical Journal Editors (ICMJE) issued a statement advocating those trials must be registered at or before the onset of patient enrollment as a condition for publication consideration [20]. Meanwhile, there were some legislative initiatives related to pediatrics [21, 22], such as in December 2003, the U.S. Congress passed a program including many provisions of the pediatric rule called PREA. In January 2007, the European Union attempted to address several unresolved issues related to the demand for medicines for children in Europe, and new regulations on pediatric medicines came into force. In this study, we found the number of clinical trial registrations about stem cell therapy in pediatric diseases has increased significantly after 2003 as well as after 2007, this phenomenon was more or less related to these incentives and regulations. Comprehensive reporting and systematic publication of clinical research results not only provided a reliable basis for the formulation of evidence-based medicine and health policy, but also further promoted the development of public health as well as clinical medicine [23] Since we found that *ClinicalTrials.gov* database and ICTRP website did not address issues, such as without truly related research results or omission in linking publications to the registration experiments, we manually searched PubMed and Google Scholar to determine potentially-relevant researches. In our analysis, of which clinical trials with primary results, the 5-year cumulative publication rate was < 40% and plenty of trials had no results published. Previous analyses reported that a large number of results were not published 2–4 years after the trials were completed [24]. Joseph S Ross et al*.* reviewed the clinical trials funded by NIH in *ClinicalTrials.gov*, their results showed that within 30 months of the trials’ completion, only 294 (46%) were published in the peer-reviewed journals [23]. Similarly, two studies indicated that around 70% of the studies had no web links for the publications of results on *ClinicalTrials.gov* [25, 26]. Except for the experiment being incomplete, there might exist a number of factors resulting in these low publication rates, including that journals tended to report only “positive” research in terms of publication bias or the researchers did not release their findings [27].

According to our analysis, the median time from registration submitted to post was 7.00 (3.00–14.50) days, and 53.9% of the clinical trials were published within 7 days of submission. In addition, for 168 trials with a detailed recorded start date, the median time difference between the trial start date and the posted date was − 26.00 (− 360.00–35.50) days, and 104 trials had begun prior to the posted date of the trial. More interestingly, 42 trials (25.0%) were posted 365 days after the start date. We also found that a large proportion of trials were released long after they were submitted. Therefore, we further investigated the distribution of time intervals between the trial start date and the submission date. Overall, the median time difference between the time intervals was − 8.50 (− 310.50–67.00) days, of which 109 trials (54.5%) had already started prior to the trial submission. *ClinicalTrials.gov* registration was established to make research more transparent and reduce publishing bias. Meanwhile, providing researchers with timely research information is the main task of the registration platform. All trials with preliminary conclusions should be encouraged to publish their findings. Both implementers and platforms should strive to make all detailed registration information and research results publish immediately in the *Clinicatrials.gov* registry [25].

Most of the included trials were interventional because of the therapeutic purpose. A well-designed clinical trial, including appropriate randomization methods, reasonable masking, treatment allocation, and accurately based sample sizes, not only efficiently decreased biased treatment comparison but also facilitated evidence-based practice [28]. Objectively, it was much more difficult to recruit children in trials than adults, and most eligible trials remained in phase 1 or phase 2, resulting in a sample size of less than 50 for the great part of studies we included. Of the 736 pediatric trials of oral and intravenous administration published between 1996 and 2002, only 38% had more than 100 samples, according to a literature search published on MEDLINE [29]. Small sample sizes might be too slight to be assessed and further lead to a lack of power [30]. DMC was a group appointed to monitor the safety and scientific integrity of human research interventions and make recommendations to sponsors regarding the effectiveness, harms, or ineffectiveness of terminating trials [31]. Compared to some studies that established DMC below fifty percent, our results seemed to be slightly superior [33]. Underpowered trials may lead to uncertain results and clinically relevant results, including adverse reactions, could not be found [34]. Single-center clinical trials were prone to own a variety of biases, such as local effect bias, selection bias, publication bias, and so forth [35]. On the contrary, large-scale multicenter research was conducive to increasing patient recruitment, speeding up research progress, and improving the effectiveness of research [36]. Obviously, in our study, especially in non-European and American countries, single-center clinical trials still exceeded 50%. Therefore, it was necessary to expand the clinical research of multicenter with uniform standards for the stem cell treatment of childhood diseases. Randomization was commonly-considered as a symbol of high-quality clinical trials [38]. Bias could be reduced through randomization and blindness, which increased the reliability of research evidence [25]. Compared to one clinical research conducted in the adults [39], most interventional studies in this paper did not report allocation or open label. All these suggested that the clinical research design in the field of children’s stem cell therapy needed to be improved urgently.

The benefit of using autologous cells was that immune rejection was not necessary to be considered, while the disadvantages included quantitative limitations and long preparation period. In contrast, allogeneic cells could be prepared readily and quantitatively on demand, but quality control was essential due to the immune rejection [40]. Our results indicated that, within the time period before 2003 and 2003–2007, the proportions of autologous and allogeneic cells were inverse. As time came after 2007, the trials using autologous and allogeneic cells were almost the same number, while from 2018 to mid-2021, the number of clinical studies using allogeneic stem cells surpassed the number of studies using autologous stem cells, which might be ascribed to that the researchers around the world had not reached a united opinion about which one was the better choice for stem cell therapy usage. Embryonic tissue; fetal tissue, such as the fetus, placenta, amniotic fluid, and umbilical cord, as well as some certain parts of adult tissue make up the main source of stem cells [41]. Hematopoietic stem cells, which were the most radically-characterized tissue-specific stem cells so far, have been investigated experimentally for more than fifty years [42]. In recent years, MSCs have been more and more widely-used in disease treatment. The reasons why MSCs have received widespread attentions were that, compared to other stem cells, MSCs did not raise ethical concerns and had a limited risk of developing tumors [43]. Our analysis suggested that most of the stem cells used in pediatric diseases were HSCs, followed by MSCs. And MSCs showed a gradual increasing trend according to the time series analysis results. According to our findings, in clinical trials of stem cells for pediatric diseases, the most common sources of stem cells were from umbilical cord blood and bone marrow. This phenomenon could be connected with the main sources of HSCs and MSCs [44–46].

## Conclusion

Stem cell therapy, as an emerging approach of rapid expansion for disease treatment, has aroused widespread research enthusiasm all over the world. The clinical trial registration platform provides a convenient way for researchers to keep abreast of the progress of research in this field. As an important member of the research in the field of life and health, the special group of children deserve special attention. We conducted a comprehensive and systematic analysis of stem cell treatments for childhood diseases registered on *ClinicalTrials.gov* and the ICTRP portal site. The following conclusions have been drawn:

1. Clinical trials in the field of stem cell treatment of pediatric diseases were inadequate in quantity and quality. Consequently, larger, multicenter, and better-designed trials were urgently needed.
2. More investment was needed, such as, to facilitate the participation of industries as collaborators, especially in the clinical trials sponsored by hospitals and universities. Clinical trials of biological products and stem cell therapy were needed to realize the early date transformation of clinical trial results, so they could benefit children as soon as possible.
3. The registration platform also needed to urge the registers to enhance the integrity of their clinical data and to upgrade the audit efficiency of registration trials, as well as joint researchers’ own efforts to shorten period of the study starting time and reporting of the final research results, so as to fully exploit advantages of the registration platform.

## Declarations

### Availability of data and materials

Data will not be shared for some privacy reasons.
